# Associations between the COVID-19 Pandemic and Hospital Infrastructure Adaptation and Planning—A Scoping Review

**DOI:** 10.3390/ijerph19138195

**Published:** 2022-07-04

**Authors:** Costase Ndayishimiye, Christoph Sowada, Patrycja Dyjach, Agnieszka Stasiak, John Middleton, Henrique Lopes, Katarzyna Dubas-Jakóbczyk

**Affiliations:** 1 Europubhealth, Institute of Public Health, Faculty of Health Sciences, Jagiellonian University Medical College, 31-008 Krakow, Poland; 2 Health Economics and Social Security Department, Institute of Public Health, Faculty of Health Sciences, Jagiellonian University Medical College, 31-008 Krakow, Poland; christoph.sowada@uj.edu.pl (C.S.); katarzyna.dubas@uj.edu.pl (K.D.-J.); 3 Health Care Management, Institute of Public Health, Faculty of Health Sciences, Jagiellonian University Medical College, 31-008 Krakow, Poland; patrycja.dyjach@student.uj.edu.pl (P.D.); agnieszka.stasiak@student.uj.edu.pl (A.S.); 4 Association of Schools of Public Health in the European Region (ASPHER), 1150 Brussels, Belgium; johnmiddleton@phonecoop.coop (J.M.); henrique.lopes@sharen.pt (H.L.); 5 Comité Mondial Pour Les Apprentissages tout au Long de la vie (CMAtlv), Partenaire Officiel de l’UNESCO, 75004 Paris, France

**Keywords:** hospital infrastructure, repurposing, adaptation, planning, COVID-19, SARS-CoV-2, health emergency, digital health, healthcare

## Abstract

The SARS-CoV-2 pandemic has put unprecedented pressure on the hospital sector around the world. It has shown the importance of preparing and planning in the future for an outbreak that overwhelms every aspect of a hospital on a rapidly expanding scale. We conducted a scoping review to identify, map, and systemize existing knowledge about the relationships between COVID-19 and hospital infrastructure adaptation and capacity planning worldwide. We searched the Web of Science, Scopus, and PubMed and hand-searched gray papers published in English between December 2019 and December 2021. A total of 106 papers were included: 102 empirical studies and four technical reports. Empirical studies entailed five reviews, 40 studies focusing on hospital infrastructure adaptation and planning during the pandemics, and 57 studies on modeling the hospital capacity needed, measured mostly by the number of beds. The majority of studies were conducted in high-income countries and published within the first year of the pandemic. The strategies adopted by hospitals can be classified into short-term (repurposing medical and non-medical buildings, remote adjustments, and establishment of *de novo* structures) and long-term (architectural and engineering modifications, hospital networks, and digital approaches). More research is needed, focusing on specific strategies and the quality assessment of the evidence.

## 1. Introduction

The 2019 coronavirus pandemic, also known as COVID-19 [1], is often used as an example of a large-scale public health emergency. It has caused unprecedented hardship in the hospital sector around the world. Unlike previous pandemics [2], daily COVID-19 cases crossed the grim milestone of surpassing infrastructure capacity in hospitals worldwide. Hospitals faced an unpredictable rise in COVID-19 daily infections [3,4], putting them under extreme strain due to a lack of sufficient infrastructure and equipment [5].

In order to respond, the overall goal was primarily to support failing hospital capacity. For this goal, a technique was successfully executed in China [6], where Fangcang shelters were initially constructed in Wuhan to help formal hospitals offer care to diseased and potentially exposed patients, was practically supported [7]. Thus, Fangcang hospital facilities have been adopted in several other countries. Some countries, particularly in Europe and the United States, did not use exactly the same facilities as Wuhan (hospitals primarily for isolation), but temporary field hospitals were welcomed and extensively used [8,9]. At the same time, considerable efforts were being made behind the scenes to develop new strategies to ensure adequate public healthcare infrastructure and workplace capacities [9]. Hospitals have repurposed and reallocated internal space and redeployed resources to manage COVID-19 patients. Countries, such as Italy and Spain, discharged many patients from hospitals to their homes and postponed non-critical treatment and elective procedures [8,10]. Also, Spain, Italy, and Portugal have converted rehabilitation facilities, hotels, public halls, and other suitable facilities into temporary COVID-19 hospitals, clinics, and laboratories [8,9]. The latter strategy was proposed in a reflection on “fever hospitals” [11,12], which were once used to isolate and treat people suffering from infectious illnesses [11].

Importantly, the COVID-19 pandemic has awoken us to the necessity of establishing health facilities that can handle large numbers of patients requiring thorough medical care in an emergency. Our general goal was to identify and map the available literature on the COVID-19 pandemic’s relationships with hospital infrastructure adaptation and capacity planning around the world. Our five specific objectives were (1) to identify relevant publications dealing with hospital infrastructure during the COVID-19 pandemic, (2) to map out the characteristics of the studies, (3) to examine reported solutions, (4) to identify research gaps for future research, and (5) to map recommendations to improve the planning of hospital capacity to manage large-scale health emergencies in the future.

To the authors’ best knowledge, no study has been conducted on the long-term vision and adaptations of hospital infrastructure in the context of COVID-19 or other rapidly spreading and large-scale infectious disease emergencies. Some related reviews, but without necessarily focusing on the COVID-19 pandemic, include a scoping review by Luke et al. [13], which has focused on assessing safer hospital infrastructure in the event of social and natural disasters. A few reviews [14,15,16,17] focused on hospital capacity during COVID-19, but they were only concerned with short-term adaptations and were published in 2020, focusing on early pandemic publications and, of course, included a small number of studies.

## 2. Materials and Methods

**Figure 1:**
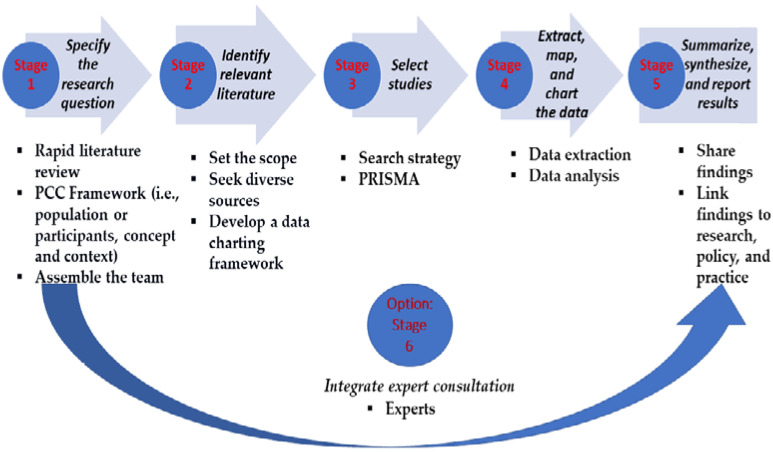
Steps of Extended Scoping Review based on Teare and Taks [18].

We reviewed the literature using Teare and Taks’s six-step scoping review framework [18] (Figure 1), which was extended from Arksey and O’Malley [19]. The protocol of the study was pre-registered with the Open Science Framework [20] in December 2021.

The “Preferred Reporting Items for Systematic Reviews and Meta-Analyses extension for Scoping Reviews (PRISMA-ScR) checklist [21]” helped structure reporting of the results.

### 2.1. Definition of Research Questions (RQs)

Five questions encoded with RQ1 to RQ5 were established to guide our review:

- RQ1: What were the general characteristics of the study (authorship/title; date of publication; studies focusing on specific countries or with a regional or global approach)?
- RQ2: What was the design of the study?
- RQ3: What was (were) the objective (s)/type of association (s) assessed?
- RQ4: What was (were) the proposed solution(s), adaptation(s), concept/theoretical framework(s), or planning?
- RQ5: What limitations have been specified?

### 2.2. Identification of Studies

**Figure 2:**
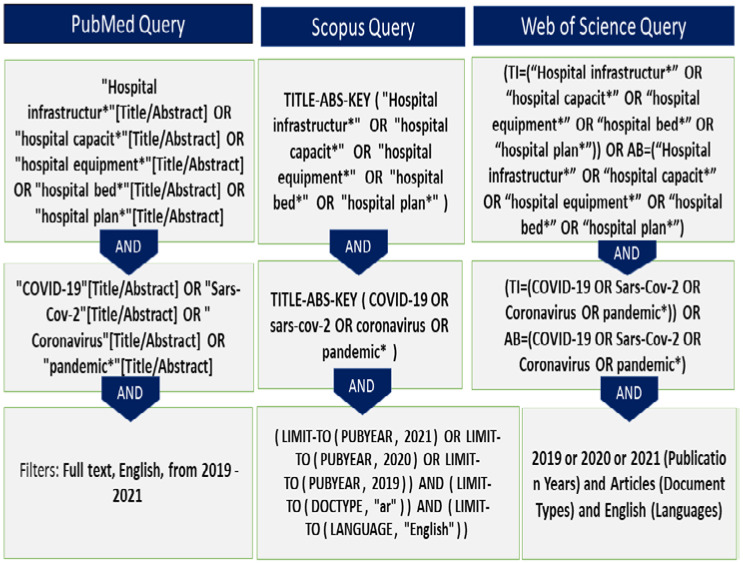
Search strategy query in three databases: PubMed, Scopus, and Web of science.

Three databases (MEDLINE via PubMed, Web of Science, and Scopus) were searched using key terms (Figure 2) for the two core concepts: “hospital infrastructure” and “COVID-19”.

The publication dates limitation was set between December 2019 and December 2021. This period allowed for extensive coverage of relevant literature because the COVID-19 outbreak started in December 2019 in China (Wuhan) [22], while most other countries recorded their first cases in 2020 [23,24]. In addition, the reference list of the included studies was scanned, the grey literature was hand-searched thoroughly, and relevant organization websites were screened (Supplementary Materials, Table S1).

### 2.3. Selection of Studies

The records of the three databases were deduplicated via Mendeley reference manager and loaded into Rayyan Desktop [25] for title/abstract and full text screenings. The screening was carried out according to the predefined inclusion and exclusion criteria (Table 1). As suggested by researchers, such as Lavac et al. [26], the selection of studies required at least two researchers to work independently, but meetings occurred on several occasions to resolve identified problems. A random 10% sample of retrieved studies was independently investigated, and the researchers reached an agreement of 95%; thus, a third researcher was not appointed to make the final selection. Due to the high agreement rate, the remaining records were screened by one researcher (the lead author of this study).

### 2.4. Extraction of Data and Charting

An excel spreadsheet specifically designed for research questions was used to extract data (Supplementary Materials, Table S2). Codes were assigned to the extracted information to help with later analysis where applicable.

### 2.5. Results: Collating, Summarizing, and Reporting

We followed the three-step approach proposed by Levac et al. [26] for researchers to conduct scoping investigations in a uniform manner. We first analyzed the data, then reported the results, and finally applied meaning to the findings. We conducted a descriptive quantitative summary and a theme analysis. The reporting was based on the total number of studies included, which were presented using both simple tables and graphical presentation. The implications of the results were considered in the wider context.

### 2.6. Consultation

Consultation is not a mandatory step in conducting scoping reviews [19], but it has been included to improve the scientific rigor [26]. The purpose of consultations, according to Arksey and O’Malley [19], is to obtain additional information, opinions, significance, and relevance to the research. We consulted experts in the field of health policy. We also presented preliminary results during a scientific webinar hosted by the Institute of Public Health at Jagiellonian University.

## 3. Results

### 3.1. General Overview of the Findings

#### 3.1.1. Number of Publications

**Figure 3:**
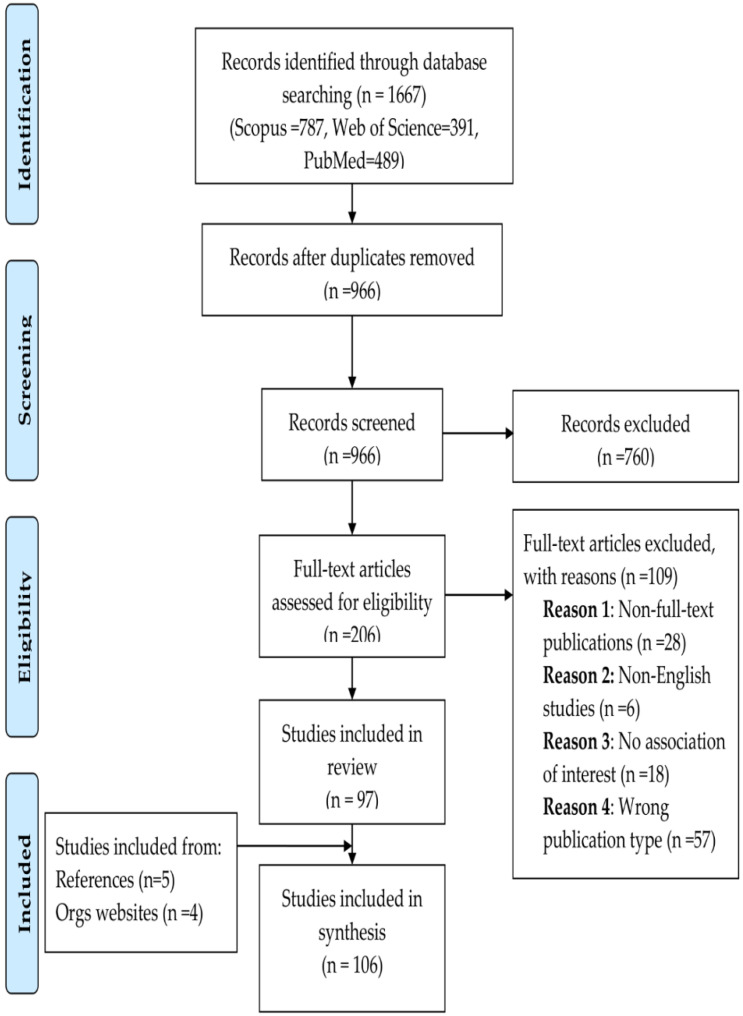
PRISMA flowchart of the results.

We found 1,667 records in the databases (Scopus = 787, Web of Science = 391, PubMed = 489; Tables S3–S5 in the Supplementary Materials). All records were successfully imported, and 966 remained after deduplication. A total of 206 studies reached the full text analysis stage, of which 97 were included in the final analysis. There were four reasons for the exclusion of the other 109 studies: first, 28 non-full-text publications; second, six non-English-language studies; third, 18 were incompatible with the association of interest; and fourth, 57 incorrect publication types (letters to editors, commentaries, etc.). We added nine new studies, five of which were from reference list screening and four from website sources. Overall, we included 106 papers in total (Figure 3 is the PRISMA flowchart, and a full list of papers included is in the Supplemental Materials, Table S6).

#### 3.1.2. Type of Publications

**Figure 4:**
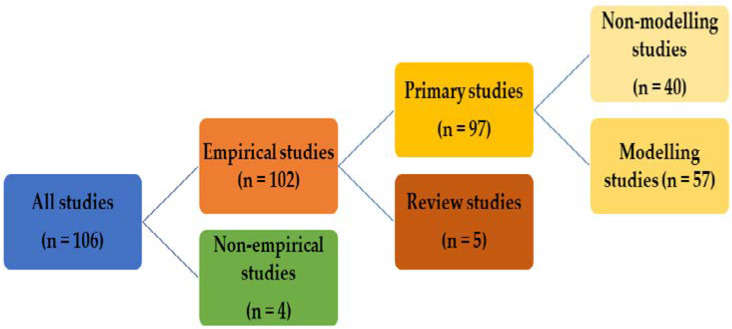
Subsets of the 106 studies included in the study.

All 106 studies included in the synthesis were divided into empirical studies (*n* = 102) and non-empirical studies (*n* = 4) (Figure 4). Empirical studies were further divided into primary studies (*n* = 97) and review studies (*n* = 5). The primary studies were finally classified as non-modeling studies (*n* = 40) [6,7,9,27,28,29,30,31,32,33,34,35,36,37,38,39,40,41,42,43,44,45,46,47,48,49,50,51,52,53,54,55,56,57,58,59,60,61,62,63] that focused on issues related to hospital infrastructure adaptation and planning or as modeling studies (*n* = 57) [64,65,66,67,68,69,70,71,72,73,74,75,76,77,78,79,80,81,82,83,84,85,86,87,88,89,90,91,92,93,94,95,96,97,98,99,100,101,102,103,104,105,106,107,108,109,110,111,112,113,114,115,116,117,118,119,120] that focused on predicting the number of hospital beds needed.

The five reviews focused on proposed adjustments to the health system during COVID-19 (Supplementary Materials, Table S7) and the number of included studies ranged from 6 to 52. A review by Sim et al. [121] looked at how COVID-19 and the subsequent pressures due to scarcity of health resources, workers, and infrastructure have promoted the acceptance and speed of digitalization. Another review by McCabe et al. [14] focused on how NHS England ensured adequate capacity to provide care to COVID-19 and other patients. The other three reviews by Rees et al. [15], Ravaghi et al. [17], and Klein et al. [16] focused on studies on the modeling of hospital surge capacity and planning. Finally, among the four non-empirical studies were three technical reports [122,123,124] on COVID-19 response strategies and hospital infrastructure, as well as a chapter in the book [125] on hospital infrastructure and the impact of various mitigation strategies in the context of the COVID-19 outbreak.

#### 3.1.3. Geographical Distribution and Publication Period of Empirical Studies

**Figure 5:**
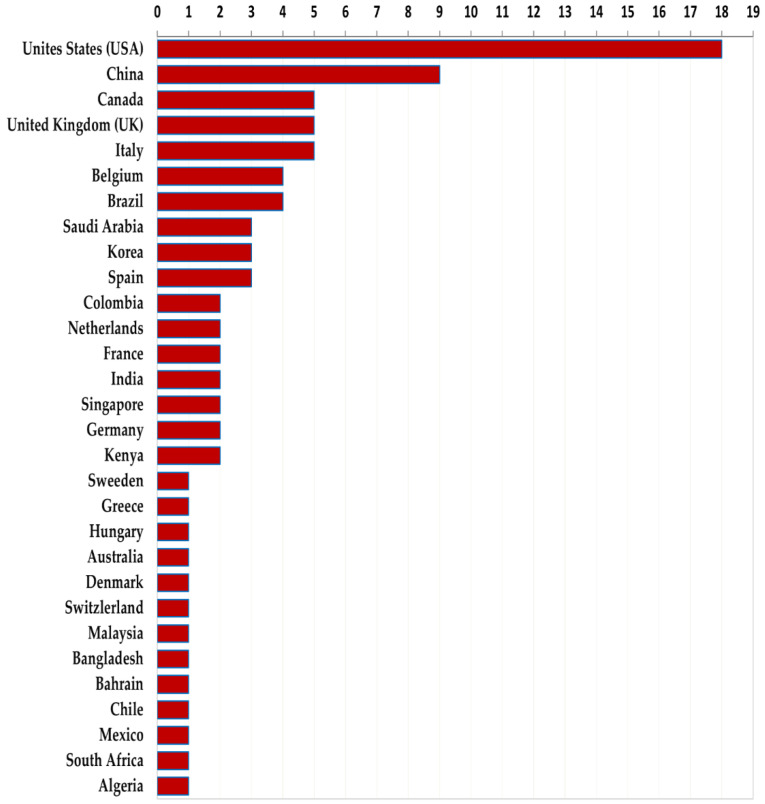
Number of studies per country of the research.

The 97 primary studies were conducted in 30 countries worldwide (Figure 5), and the majority (74/97, or 76.3%) were from high-income countries. Moreover, 87.6% (85/97) focused on individual countries, while 12.4% (12/97) were multi-state based. More than one-third (31.8%, or 27/85) of all single country-based studies were conducted in the United States (*n* = 18) and China (*n* = 9).

**Figure 6:**
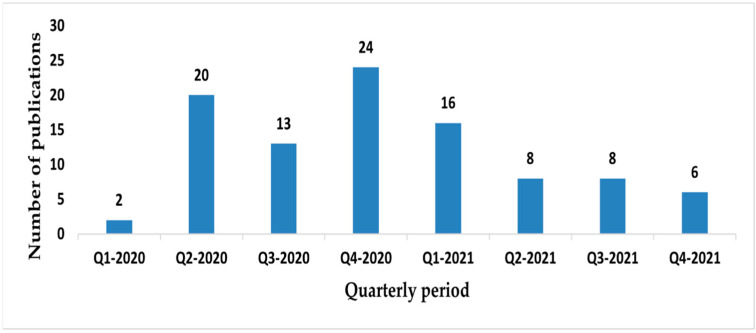
Published studies for each quarterly period, between January 2020 to December 2021.

Of all primary studies, 73.3% (73/97) were published in the first year of the pandemic (March 2020–March 2021) (Figure 6).

### 3.2. Empirical Studies on Hospital Infrastructure Adaptation

Of non-modeling empirical studies, 47.5% (19/40) investigated the influence of the COVID-19 pandemic on hospital infrastructure (COVID → hospitals), 35.0% (14/40) evaluated COVID-19 management and available infrastructure (hospitals → COVID-19), and 17.5% (7/40) assessed both perspectives at the same time (COVID-19 ↔ hospitals). The shift from a physical monitoring strategy to a camera-based monitoring technique in the intensive care unit (ICU) for infection control would be an example of the first viewpoint (i.e., COVID → hospitals), while the example for the second one (i.e., hospitals → COVID-19) would be to transfer patients within the hospital networks.

The strategies aimed at adapting hospital infrastructure that were described in the included studies can be divided into short- and long-term. The short-term ones sought to relieve immediate pressure on hospitals without necessarily being sustained throughout or beyond the COVID-19 pandemic. They were neither definitive nor appropriate alone for consistent use once the care demand could be handled within normal hospital capacity. The opposite is true for longer-term strategies, and they were particularly suggested as an important part of hospital planning. Overall, 32 of the 40 studies included both short- and long-term strategies; eight studies were solely focused on short-term adaptations, and none were solely focused on long-term strategies.

**Figure 7:**
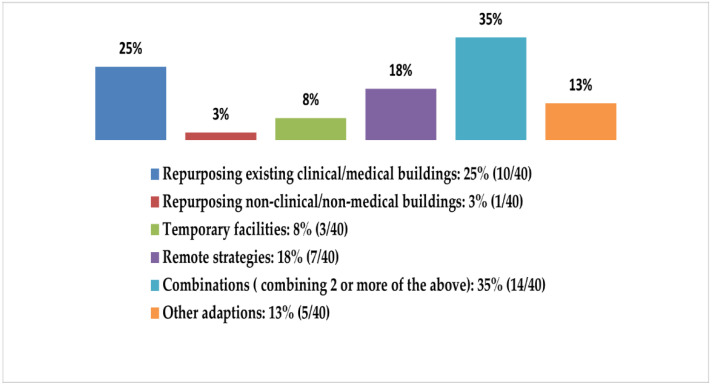
Six broad categories of short-term strategies used to adapt a hospital’s capacity during the COVID-19 pandemic.

The short-term adaptations (Figure 7) expanded the hospital infrastructure capacity by employing at least one of the six broad strategies as follows: 25% (10/40) focused only on repurposing existing medical buildings or their spaces (converting pediatric rooms into ICU beds, converting hospital parking lots into ICUs, etc.), 18% (7/40) focused only on remote strategies, 8% (3/40) were only temporary/movable facilities (makeshifts, field hospitals, etc.), only one focused on repurposing non-clinical or non-medical buildings (training centers, malls, and schools, for example), and 35% (14/40), which is the majority, focused on a combination of two or more of the aforementioned strategies. The remaining 13% (5/40) were simultaneous and/or supplementary adaptations, such as the cancellation of non-elective surgeries, transferring patients within hospital networks, and changing ICU surge guidelines, among others.

Repurposing a hospital was either complete or focused on one or a few sections of a hospital. The outpatient department (OPD), emergency departments (EDs), and ICUs were the top three sections of the hospital’s infrastructure that underwent adaptations (Table 2). Hospitals primarily focused on increasing ICU bed capacity, either directly (within ICU areas) or indirectly (within and outside of a hospital’s boundaries).

*De novo* structures included the installation of new buildings and the use of temporary facilities (makeshifts, field hospitals, Fangcang hospitals, military hospitals, and so on). These structures played at least one of the functions listed in Table 3.

Finally, 18% (7/40) of the studies reported remotely based strategies, such as through websites, call centers, and other forms of tele-technologies. Most of the research indicated that using digital health to adapt to the demand for health care in hospitals was the best way to go. “With COVID-19, we’ve reached a new level of crisis response…”, said Seema Verma, Administrator of the Centers for Medicare and Medicaid Services (CMS) in the United States. “CMS is leveraging the latest innovations and technology to assist health care systems that are facing significant challenges in increasing their capacity”, he added [127].

Of the studies focused on long-term strategies, 37.5% (15/40) were interested in a mix of architectural, engineering, and construction changes (e.g., planning of hospital parking spaces that can be converted into emergency facilities; public facilities that can easily be converted to medical facilities; and so on). Another 42.5% (17/40) described other plans such as accelerating digital technology (e.g., strengthening digital health or its related uses, for example, strengthening the hospital-at-home strategy, etc.), and building hospital networks (both public and private), as well as adaptive and complementary investment measurements to improve surge capacity.

### 3.3. Empirical Studies Predicting the Number of Hospital Beds (Modeling Studies)

**Figure 8:**
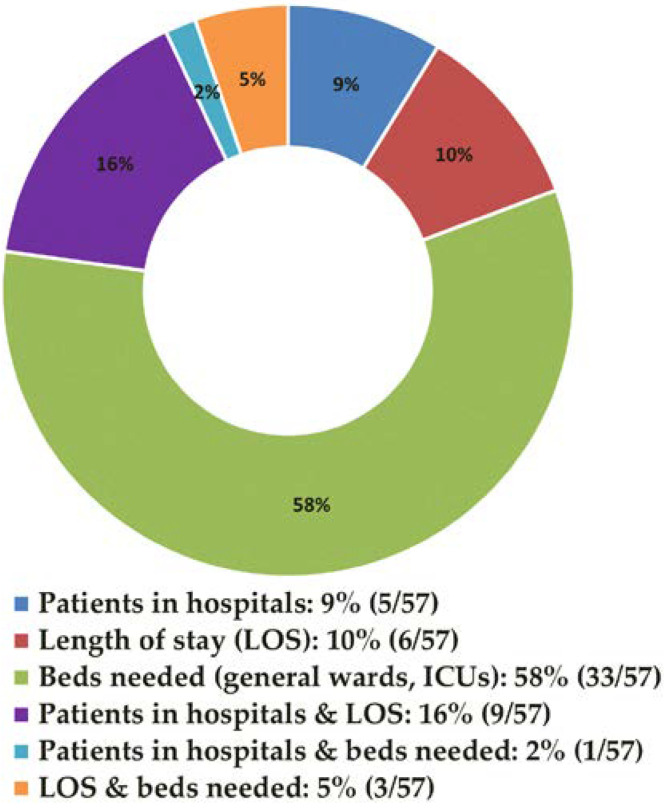
Major areas of focus of modeling studies to predict hospital capacity.

Of the 57 modeling studies, 48 modeled hospital capacity in individual countries, whereas 15.8% (9/57) included at least two countries. Researchers employed neural networks, regression models, time series, and other predictive modeling techniques. The variables most often included in the models were patients’ age, sex, and number of beds (in general wards and ICUs), deaths, discharges, and new cases. Most of these studies (58%, or 33/57) modeled the number of beds required in general hospital wards or intensive care units, and frequently both general and ICU beds at the same time; 9% (5/57) modeled the number of patients in hospitals; and finally, 10% (6/57) modeled the length of stay (LOS) in hospitals. A few of them incorporated two or more of the prediction models (Figure 8).

The studies showed a number of implications that can be divided into nine categories as follows: (1) predicting the ICU, hospital length-of-stay and bed demand; (2) hospital infrastructure’s estimation of the size of the epidemic, forecasting the early course of a pandemic, its progression, and its severity overtime; (3) predicting the peak time and the number of infectious cases at the peak; (4) the impact of the shortage of PPEs on the spread of the disease; (5) examining the possible impact of a full lockdown, if implemented; (6) predicting the excess demand for services that a pandemic can generate; (7) allowing for the simultaneous visualization of the epidemic’s speed (new hospitalizations per day) and acceleration (daily growth rate) with a forward-thinking approach to ICU occupancy and informing capacity strain preparations; (8) preventing hospital overcrowding by projecting when capacity may need to be increased and planning ahead of time for referral or other mitigation strategies; and (9) geocoding to locate nearby hospitals during crisis management.

The limitations of the models listed by the authors fall into at least one of the following five classes: (1) under-or over-estimation of the time-to-event; (2) ambiguity resulting from the inability to deduce transmission parameters and the inherent construction of a model in various epidemic compartments; (3) difficulties in incorporating uncertainty, more transition states to model the disease’s drivers, the determinants of disease severity and social determinants; (4) failure to consider seasonal factors; and (5) isolation conditions and the effect of self-isolation of symptomatic cases.

## 4. Discussion

### 4.1. Summary of the Results

**Figure 9:**
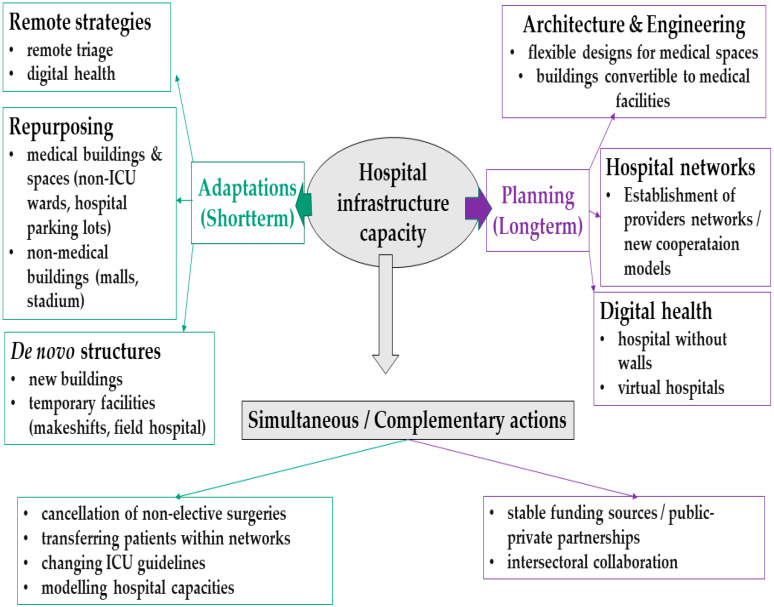
Graphical representation of hospital infrastructure adaptation and planning during the COVID-19 pandemic.

Our review included 106 publications, 102 of which were empirical studies, and four of which were technical reports. The included studies came from 30 countries worldwide, with the majority being high-income countries. The majority of studies (73.3%) were published within the first year of the pandemic (March 2020–2021). There were 97 primary empirical studies, 40 of which focused on hospital infrastructure adaptation or planning and 57 on estimating the number of required hospital beds. The identified strategies can be divided into short-term and long-term (Figure 9). The former helped reduce immediate pressure on hospitals, but they were not meant to last throughout the pandemic or beyond. Long-term strategies are the opposite of a quick fix and focus on planning hospital infrastructure for the future.

The strategies outlined exclusively deal with hospital infrastructure; nevertheless, other concurrent and complementary activities were also required in response to COVID-19 crisis management. Non-urgent procedures were canceled, patients were transferred within hospital networks, ICU surge guidelines were adjusted, etc. The subsections that follow concentrate on hospital infrastructure adaptations and planning strategies.

### 4.2. Hospital Infrastructure Adaptation Mechanisms

Our study showed that while primarily seeking to expand hospital infrastructure capacity during the COVID-19 pandemic, the general idea was to look at ways to repurpose existing facilities, install new structures, and employ various remote strategies. These findings are consistent with those of other studies, notably Witcher [34], who shows that the United States has rapidly increased the number of beds to serve a health care system that was expected to stretch its limits by converting a number of medical and non-medical buildings, as well as through the construction of new buildings.

Hospital repurposing ranged from partial to complete. For example, Raith et al. [44] discuss repurposing a neurocritical care unit in the UK to manage severely ill COVID-19 patients, and Deep et al. [126] describe the experience of pediatric units at two tertiary hospitals in London and New York running a hybrid model of pediatric and adult critical care during the 2019 Coronavirus disease. The complete repurposing is also covered by a few researchers, such as Chen et al. [33], who showed how Zhejiang University Hospital (FAHZU) accomplished the transformation from a general hospital to an infectious disease hospital in response to the coronavirus epidemic, and Kim et al. [46], who documented the experiences of converting a general hospital into a COVID-19 hospital (Keimyung Daegu Dongsan University Hospital, in Daegu, Republic of Korea).

Attempting to satisfy the demand for beds also meant identifying existing non-medical buildings that might be suitable for use. Training halls, exhibition centers, malls, and schools, among other things, have been set up to temporarily relieve pressure on hospitals. Quite often, when converting non-medical buildings into medical structures, new structures were also erected, such as tents. This creation of temporary hospital capacity was extensively studied in the included studies. For example, Jefferson and Heneghan [11] show that temporary facilities and field hospitals became necessary during the first wave of the pandemic, while Budhiraja et al. [128] say that those facilities were likely needed even more for the second wave of the pandemic. These kinds of adaptations, however, might be highly dependent on the localization characteristics (e.g., level of urbanization and the availability of convertible spaces).

Importantly, temporary facilities display three chief features that make them very crucial to managing health emergencies growing rapidly and on a large scale like COVID-19 [6]. Firstly, they are built in a short period of time. Because they are based within an existing physical infrastructure, they can be constructed quickly. The conversion process chiefly comprises some space reorganization as well as the procurement and setup of beds, equipment, and supplies to enable shelter, care, surveillance, and triage. The latter are some of their main characteristics and functions, as claimed in several other studies, such as Chen et al. when studying Fangcang hospital [6]. The second attribute is large scale, which usually results in more beds. We found that large public venues, such as stadiums, convention centers, and conference halls, were among the non-medical facilities that were converted. It is easily understandable that making use of large-scale public facilities (when converted to hospital operations) would result in a significant boost in terms of hospital capacity (beds, space, etc.). For example, in response to the COVID-19 pandemic, the London National Health Service (NHS) Nightingale hospital was accomplished in just nine days thanks to the vast space provided by the ExCeL London Convention Centre [12,129]. This temporary hospital was opened quickly on 3 April, with the first patients arriving on 7 April 2020. Initially, it started with a capacity of 500 beds, with the ability to expand to 4000 beds. Although this NHS Nightingale in London was the first one in the United Kingdom, many others were in the pipeline in the meantime. Within two weeks, the NHS constructed the equivalent of 54 district general hospitals in terms of bed capacity [12]. The third feature is the low cost of building and running the temporary hospitals. Because transforming public spaces into healthcare facilities eliminates the need for costly new physical infrastructure, investment costs are thus modest [6]. Additionally, once the outbreak has passed, the facilities can be reconverted to their original functions, preventing inefficient long-term space utilization. This is especially a crucial consideration in highly populated areas. Moreover, because fewer nurses and doctors are needed than in typical hospitals, and since most cases require standard procedures, the operating costs might be lower.

As stated previously, converting and repurposing medical and non-medical building facilities, as well as establishing *de novo* structures, undoubtedly contributed to the expansion of capacity and relief of pressure in traditional hospitals. In terms of sequencing the response strategies, non-medical facilities are less likely to be converted before changes in regular hospitals are conducted. With patients occupying and surpassing regular hospital capacities, the first step would usually encompass freeing up more space and adding beds within hospital systems’ existing areas. For example, as a result of the rapid increase of COVID-19 patients in hospitals, on 17 March 2020, NHS England (NHSE) issued an order directing all hospitals to free up bed capacity by releasing as many non-critical patients as possible and to halt all planned elective surgeries by 15 April [130]. These measures aimed to free up 30,000 or more of the 100,000 general and acute beds in the English NHS, supplementing them with an additional “all available” bed capacity [130]. This is consistent with the results of our study, in which the cancellation of non-urgent surgeries was one of the other simultaneous/supplementary adaptations to increase hospital infrastructure capacity to accommodate patients. Like the United Kingdom, other countries, such as Italy and Spain, discharged many patients from hospitals to their homes and postponed non-critical treatment and elective procedures [8,10].

The next step would be to set up temporary facilities, such as tents, on the hospital grounds and to identify any vacant hospital spaces. Some hospitals used parking spaces for the latter [47]. The last step would be creating new hospital areas, for example, by using hotels, convention centers, and municipal parks or by using transportable and makeshift facilities. Some European countries, such as Spain, Portugal, and Italy, converted rehabilitation facilities, hotels, public halls, and other suitable facilities into temporary COVID-19 hospitals, clinics, and laboratories [8,9].

We discovered that there are pros and cons to using temporary facilities. We found that temporary facilities could serve patients with mild to moderate symptoms and could only perform a few set activities, such as triage, speedy referral, and so on. This indicates that these facilities, regardless of their purpose, fall short of typical hospital requirements. This is in line with other researchers such as Chen and Zhao [131], who found that many converted buildings’ heating, ventilation, and air conditioning systems are incapable of recreating hospital ICU beds. The makeshift hospital wards are probably more useful for isolation and for providing medical care for patients who are mildly sick. Converted sports areas and hotels might serve as quarantine centers for patients with milder symptoms. “The most important thing to understand is you can’t be exactly a hospital with all the compliance issues and the standards and the airflows when you’re putting 500 or 600 patients in the open areas in a convention center”, says Stan Shelton, vice president of organizational development and client engagement leader at HKS Architects, “you have a lot of hospital-like activities, and you will absolutely be providing health care in those spaces, but it’s not going to be exactly like a hospital” [132].

The other adaptation (different than repurposing buildings and establishing *de novo* structures) was to free hospital capacity by moving as many patients as possible to remotely based care. This was observed in 18% (7/40), while its combination with other tactics was reported in 35% (14/40) of studies. In fact, everything that could be moved remotely was moved to free more hospital capacity to care for critically ill patients. These could mostly apply to services offered by the OPD, as well as other non-urgent care. The remote strategy could be coupled with already existing goals to move care closer to the patients at home, “hospital at home” or telehealth. Meanwhile, some remote strategies, such as e-health, are implicated in both short-and long-term applications. For example, quickly shifting to providing outpatient consultations online during an outbreak may appear to be a short-term solution, but if sustained for daily activities in the future hospital, it becomes a long-term strategy.

The COVID-19 outbreak has also sparked the creation of some innovations integrating both concepts: hospital-at-home, telecare, and in-patient care options. The one we are interested in sharing is the “hospital without walls”. On 25 November 2020, the US Centers for Medicare and Medicaid Services (CMS) unveiled “Hospitals Without Walls” [127], as a comprehensive approach to increasing hospital capacity. This strategy entailed hospital-at-home units, ambulatory facilities, and inpatient service options. These flexible options made it possible for home health care with the help of telehealth, as well as serving as ambulatory surgery centers and providing inpatient treatment services, which allowed patients to stay longer than usual when needed. By doing so, routine hospital procedures were being performed outside of hospital settings, thus allowing hospitals to maintain adequate capacity and focus on the increased demand for care caused by the COVID-19 pandemic. In addition, a variety of outpatient options were available, each tailored to the needs and resources of the local community. The goals of the outpatient alternatives were translated into a range of treatment intensity levels, ranging from providing shelter to patients whose residences did not permit quarantining to treating patients with mild symptoms. As a result, this “hospital-without-walls” strategy has aided US hospitals in providing patient care outside of traditional hospital settings [127,133], and it has benefited COVID-19 care provision for other vulnerable groups such as the homeless, black US residents of inner cities, and economically challenged communities [133]. The latter additions are in line with our findings, which revealed that offering basic care to non-ill patients, shelter, and food were among the *de novo* facilities’ primary functions.

Ultimately, although not an infrastructural adaptation itself, hospital capacity modeling was an important complementary solution. Modeling the number of patients in hospitals, length of stay, and the number of beds provides information about the required capacity in the hospital infrastructure, which drives its adaptation and planning.

### 4.3. Hospital Infrastructure Planning

COVID-19 has shown that existing healthcare facilities need to be upgraded to meet future demand if another outbreak strikes. Many existing hospitals face challenges in making improvements [134], as their designs are inflexible and difficult to change. However, the literature also provides good examples of hospitals that were created with the intention of being easily adaptable for providing care in emergency situations. “Rambam parking lot will serve as hospital”, Siegel-Itzkovich of the Jerusalem Post wrote in 2012, “Medical center’s new underground parking facility will double as 2000-bed emergency hospital for North” [135]. They reported this in 2012, and it became true several years later during the COVID-19 pandemic. This Rambam Hospital in Haifa, Israel, has converted its subterranean parking garage into a well-suited 2000-bed hospital [136]. Rambam Hospital was designed with digital infrastructure, filtration, and hospital-grade ventilation in mind. The hospital had a large stockpile of medical equipment, like oxygen tanks, beds, and dialysis machines, ready to be used at a moment’s notice. The emergency facility was constructed eight meters below sea level and is capable of generating its own electricity. Adaptability is present in all spaces. A large part of the hospital has been turned into a dedicated COVID-19 department, with its own separate elevator and ambulance entrance. Before leaving the building, the air from the rooms could be filtered. The rooms have been outfitted with telephone and video technology so that family members and medical personnel can communicate with one another, with the goal of limiting contact but still keeping connectivity.

Another hospital with flexible capacity is the Rush University Medical Centre (in Chicago) [136,137], which can boost ED and isolation room capacity as needed. After 9/11, Rush University Medical Centre was created to provide medical care for a surge in patients from large-scale catastrophes, like terrorist attacks, industrial accidents, or outbreaks. As a result of being ready, it responded quickly when SARS-CoV-2 broke out. The ED’s air handling system is divided into three sections, each with a capacity of 20 beds. The ER has 60 beds, but in the event of a crisis, each section (also known as a unit or pod) can be doubled to expand the capacity to 120 beds. Each pod can be isolated separately, as necessary, to manage more potentially contagious patients. During ordinary operations, the Rush Centre has 40 negative pressure rooms to help stop airborne infections from spreading, but within only two hours, the facility can quickly convert another wing into a negative pressure ward, increasing its isolation capacity by 32. In an emergency situation, an “emergency MASH unit” is created from the main atrium of the facility and completely disconnected from the ED. Regular patients who visit the emergency room can receive treatment in the converted atrium. The ED can be quickly adapted to accommodate high-volume screenings. It has the ability to screen an extra 100 patients each day (in addition to the usual volume of patients screened). In addition, Rush University Medical Centre also uses standards-based 5G, a technology that allows connecting a variety of applications, medical devices, telephones, and robotics equipment; enables augmented reality; increases remote monitoring capacity; allows patients to have access to the bandwidth for video chatting with their families and clinicians to deliver enhanced virtual health care; and so on [136,137].

These previous examples imply that having foresight in hospital design is essential for being more prepared in the face of evolving health-care emergencies. Research claims that most hospitals are designed for highly specialized medical purposes, which is often at odds with the necessity to build a hospital facility that can accommodate evolving and changing tasks over time. Pilosof [134] studied how architectural design techniques affect a hospital’s ability to adapt over time. Her study discovered that the design approach to suit a particular function constrained the hospital’s ability to make changes, whereas “systematic design of system separation for an unknown function,” in contrast to a “tailor-made” technique in the design for a specific purpose, was seen to support a wide range of changing care needs [134].

Similarly, as in short-term adaptations, the digital path has several implications for long-term planning if it is made an integral part of current and future hospitals. Firstly, it can play an important role in assisting physical hospitals by operating virtual hospitals. During COVID-19, virtual hospitals, also known as virtual wards, were used in many countries, but the first was created in New South Wales in February 2020 as the coronavirus hit Australia [138]. This hospital quickly adapted to providing remote care to COVID-19 patients by using pulse oximeters and armpit patches to assess body temperature. The information gathered is communicated to the virtual hospital’s care providers through an app on the users’ phones. In July 2020, the Northampton General NHS Trust in England partnered with Doccla [139], a Swedish start-up specializing in “virtual hospital care,” to remotely monitor patients recovering from COVID-19 and people with chronic conditions. In order to free up NHS capacity, patients were given wearable gadgets that monitored their vital signs (oxygen and pulse levels, for example), and their care plans could be changed at any time if there was any health deterioration. In May 2020, the UAE Ministry of Health and Prevention upgraded all of its hospital outpatient clinics to virtual clinics [140]. In May alone, over 15,000 people were being treated in virtual clinics that offered a variety of outpatient services, ranging from mental health care and physiotherapy to pediatrics and cardiology. The examples above show that virtual hospitals have the potential to change the way care is provided in the long term. As the Northampton NHS Trust experiment has shown [139], virtual hospitals can bring about extraordinary change for chronically ill patients and improve the overall level of care and follow-up that can be provided to patients away from hospitals (i.e., enhancing the hospital at home approach).

Additionally, adopting smart devices to care for chronic conditions linked with the elderly would be beneficial in offloading hospitals from the aging population [141]. In parallel, the patient’s stay in a clinic or hospital would no longer be the exclusive source of useful data for care and research purposes. Furthermore, the digital path could help hospitals more easily identify and control infections. The COVID-19 pandemic spawned a slew of innovations devoted to remote triage and infection detection [142]. If explored further, these technologies could help hospitals plan for proper infection control in the future, either by reducing nosocomial infections in high-risk hospital departments (such as the NICU and general ICU) or by improving hospital readiness for future infectious outbreaks.

The need for strong intersectoral collaboration and networks is one of the undisputable requirements to manage the COVID-19 pandemic. Clinical and non-medical personnel, such as architects and engineers, worked as an inseparable team, especially to convert the building into medical facilities and to build *de novo* structures [34,132]. In addition, we found that some hospitals were transferring patients between networks or to the private sector in order to ease the pressure. This finding is consistent with the study by Budhiraja et al. [128] on the transfer of COVID-19 patients to a network of hospitals in North India and a study by Illman [143] on NHS England requesting hospital beds and resources (doctors, nurses, and PPE) from the independent private sector for a fee. This point of swap deals in private individual hospitals or networks is a critical area that has proven to be a significant weakness in today’s health system’s ability to manage rapidly growing health emergencies, such as the COVID-19 pandemic. It also highlights the importance of strengthening public-private partnerships (PPPs) as a means to increase the preparedness and resilience of health systems, not only to prevent unnecessary deaths, but to ensure the joint resilience of health systems and a sufficient (including financial) and effective response to future health emergencies.

### 4.4. Study Limitations

A scoping review as a research method is not free from limitations. For example, it does not provide a quality assessment of the studies included [26]. Similarly, the quality was not assessed in our review. The purpose, as with all scoping reviews [19,144], was to identify, map, and describe the evidence related to the topic. Another potential drawback of our research is that we only included studies that were written in English. Despite the above limitations, we believe this study provides useful and structured information on how to adapt and plan hospital infrastructure to deal with large-scale health catastrophes.

### 4.5. Study Implications

Our study has implications for both research and policy. First, a large number of studies was published within the first year of the pandemic; this indicates that empirical evidence could be quickly developed, thus helping with adequate health policy decisions. However, a further study should focus on a more precisely defined research question and include a quality assessment of the evidence. For example, it would be interesting to assess the effectiveness of a particular strategy in terms of both securing additional capacity and impacting hospital performance. The issue of the cost-effectiveness of diverse strategies also remains open for further research. Second, the digital path is undoubtedly a potential contributor to the way hospitals managed the pandemic, and even some of the digital strategies are likely to remain in the post-COVID-19 era. There is a need for further research about the way digital health can be supported in countries, particularly those with less developed health system structures and fewer resources. Also, further research is needed to see how e-health and other digital healthcare system models can advance care access, coordination, comfortability with users (both patients and care providers), and quality of care. Third, further research is also needed to understand how hospitals can be interconnected between a mix of private and public hospitals (i.e., hospital networks) as well as how intersectoral collaboration can be strengthened.

## 5. Conclusions

The hospital sector adopted diverse strategies to adjust its infrastructure during the COVID pandemic. The short-term strategies included repurposing medical and non-medical buildings; remote adjustments; and the establishment of *de novo* structures. Long-term ones focused on architectural and engineering modifications, hospital networks, and digital approaches. Temporary facilities are necessary to help minimize negative outcomes in cases where hospitals need more capacity by performing essential functions such as triage, isolation, sheltering, and rapid transfer. However, they have some limitations, which prevent them from being similar to regular hospitals (e.g., difficulties with heating, ventilation, and air conditioning systems). For long-term solutions, these limitations should be addressed. Flexible designs, adaptable structures, and hospital annexes that can be easily adapted to serve as medical facilities are needed. System-level changes, such as new architectural guidelines, more hospital networks, and strengthened PPPs, can be helpful in responding to future outbreaks. Additionally, exploring digital technology opportunities is also needed.

## Figures and Tables

**Table 1:** Inclusion and exclusion criteria for study selection.

Inclusion Criteria	Exclusion Criteria
▪ Studies describing hospital responses to the COVID-19 pandemic, with a focus on structural and infrastructural changes to address the new challenges posed by the SARS-CoV-2 outbreak ▪ Only full text publications ▪ Full text available in English ▪ Dates limits: December 2019 to December 2021	▪ Clinical studies ▪ Studies focused on staff management (without connection to infrastructure, etc.) ▪ Studies on social pandemic management (without a focus on hospital infrastructure) ▪ Conference abstract ▪ Scientific literature that lacked an abstract or full text

**Table 2:** The areas of hospitals that were most commonly adapted during the COVID-19 pandemic and the strategies that were employed.

Reference	Area of Focus		Strategy
[126]	Adaptations to increase ICU bed capacity	(a) Within ICUs	• Use of non-operational ICU beds • Making large ICU rooms into double rooms for two or more patients. • Moving low-acuity patients to beds in the wards
[34,42,43,44,45,46,47,48,49,50,51,52,53,54,55,56,57,58]		(b) Within hospitals	• Repurposing other non-ICU beds to ICUs (e.g., EDs, ORs, etc.) • Establishing *de novo* ICUs
[34,52]		(c) Outside hospitals	• Field hospitals • Prediction models • Surveillance discharges vs. admission match
[7,28,34,43,47,48,62]	Adaptations in emergency department (ED)		• Screening patients using outside pods or tents. • Restricting the number of visitors • Separating contagious patients in waiting rooms • Infection hotspots’ designation, identification, and separation • Changes to air systems to achieve negative pressurization • Patients shift from overcrowded EDs to offsite venues for telehealth • To execute mass triage, modular or movable structures were deployed • Converting external spaces into temporary emergency care units (e.g., garages and parking spaces)
[30,40]	Adaptations in outpatient department (OPD)		• Considerable shift to telemedicine owing to: (i) temporary shutdown of outpatient clinics; (ii) use of outpatient facilities as centers for COVID-19 purposes, such as hospitalizations, treatments, isolations, testing, etc. • Adoption of the strategy known as “Hospitals Without Walls *” • Patient’s symptoms and temperature checking, preferably upon arrival to OPD; occasionally remotely

* On 25 November 2020, the US centers for Medicare and Medicaid Services (CMS) unveiled “Hospitals Without Walls” [127], an approach that at the same time entailed “hospital-at-home units”, “ambulatory facilities”, and “inpatient services options”.

**Table 3:** Essential functions of *de novo* structures.

Reference	Function of a *De Novo* Structure
[6,54,59]	Sheltering
[6,32,43,49,50,51,54,55,56,57,58,59,60,61,62,63,64,65,66,67,68,69,70,71,72,73,74,75,76,77,78,79,80,81,82,83,84,85,86,87,88,89,90,91,92,93,94,95,96,97,98,99,100,101,102,103,104,105,106,107,108,109,110,111,112,113,114,115,116,117,118,119,120,121,122,123,124,125,126,127]	Isolation
[6,36,54]	Triage
[6,54]	Basic care to non-ill patients
[6,30,54]	Frequent surveillance/ monitoring
[6,47,54,56]	Rapid transfer
[6,54]	Social interaction and necessary living
[6,54,127]	Food

## Data Availability

Not applicable.

## Supplementary Materials

The following supporting information can be downloaded at: https://www.mdpi.com/article/10.3390/ijerph19138195/s1, Table S1. Lists the organizations’ websites that were consulted, along with their addresses and dates of access. Table S2: Data extraction form for empirical papers. Table S3. Search Results in PubMed. Table S4. Search Results in Scopus. Table S5. Search Results in Web of Science. Table S6: List of all studies included. Table S7. Results of five included review studies.
