# Supplementary material for: Associations between the COVID-19 Pandemic and Hospital Infrastructure Adaptation and Planning—A Scoping Review
Source: Int J Environ Res Public Health. 2022 Jul 4;19(13):8195. doi: 10.3390/ijerph19138195 (PMC9266736; doi:10.3390/ijerph19138195)
Supplement: Supplementary file 1 [file ijerph-19-08195-s001.zip › ijerph-1760713-supplementary.pdf]

International Journal of
Environmental Research and Public Health

MDPI

Supplementary Materials

Table S1. Lists the organizations' websites that were consulted, along with their addresses and dates of access.

Name of the organization	Address of the website	Access date
▪ Organisation for Economic Co-operation and Development (OECD)	https://www.oecd.org/	15/03/2022
▪ The World Bank	https://www.worldbank.org/en/home	15/03/2022
▪ European Observatory on Health Systems and Policies	https://eurohealthobservatory.who.int/home	15/03/2022
▪ European Commission	https://ec.europa.eu/info/index_en	16/03/2022
▪ U.S. Centres for Medicare & Medicaid	https://www.cms.gov/newsroom/press-releases/cms-announces-comprehensive-strategy-enhance-hospital-capacity-amid-covid-19-surge	14/03/2022
▪ World Health Organization (WHO)	https://www.who.int/health-topics/hospitals#tab=tab_1	14/03/2022
▪ American Hospital Association (AHA)	https://www.aha.org/fact-sheets/2021-05-26-fact-sheet-ensuring-hospital-infrastructure-meets-nations-needs-today-and	14/03/2022
▪ American Society for Health Care Engineering (ASHE)	https://www.hfmmagazine.com/articles/4329-designing-the-post-pandemic-hospital	16/03/2022
▪ Centre for Disease Control and Prevention (CDC)	https://www.cdc.gov/nhsn/covid19/report-patient-impact.html	16/03/2022
▪ European Hospital & Healthcare Federation (hope)	https://hope.be/	16/03/2022
▪ Association of Schools of Public Health in the European Region (ASPHER)	https://www.aspher.org/covid-19-situation-reporting.html	16/03/2022
▪ Omnia health	https://insights.omnia-health.com/management/building-health-centres-future	16/03/2022

Table S2: Data extraction format for empirical papers

Research question (RQ)	Data extracted	Coding examples
**RQ1**: What were the general characteristics of the study (authorship/title; date of publication; studies focused on specific countries or with a regional or global approach)?	▪ Authors<br>✓ Acronym<br>✓ Full reference<br>▪ Title<br>▪ Date of publication<br>▪ Research place	• N/A<br>• Author(year)<br>• N/A<br>• N/A<br>• Year and month: e.g.: 2019 June → 2019/06<br>• 1_ research in one country (specify the country); 2_ multi country

		in same continent; 3_ multi country in different continents (international); 4_no information on research country
**RQ2**: What design was the study?	▪ Research design	• Qt_Quantitative: QtL_longitudinal; Qtc_cross-sectional; Qtm_ modelling)<br>• Ql_Qualitative<br>• Mx_Mix
	▪ Period of the study	• From (MM/YYYY) to (MM/YYYY) e.g., 01/2019 - 04/2019
	▪ Sample	• M_Multicountry; N_national; R_regional; L_local; H1_case study based on 1 hospital only
	▪ Type of hospital	• GH_general hospital, SH_specialised hospital, UH_university (teaching) hospital, NH_hospital not specified
	▪ Number of hospitals	• Number, e.g.,5<br>• Not specified (NS)
**RQ3**: What was (were) the objective(s)/type of association(s) being evaluated?	▪ Study objective (type of association being assessed)	• Impact of the COVID pandemic on the hospital infrastructure (COVID → hospitals)<br>• Association between COVID management and available infrastructure, e.g., mortality vs ICU beds (hospitals → COVID)<br>• Modelling studies on number of beds needed (hospital →bed prediction)
**RQ4**: What was (were) the solution(s) or adaptation(s) or conceptual/theoretical framework(s)/planning stated?	▪ Study results/conclusions (solution/ conceptual/theoretical framework)	• ST_ (shor-term solutions) Adaptation:<br>• ST1_Temporary facilities: e.g., Mobile makeshifts, field hospitals, etc; ST2_Repurposing non-clinical/non-medical buildings: e.g., training centers, malls, schools.; ST3_Repurposing existing clinical/medical buildings, e.g, converting pediatric rooms into ICU beds); ST4_Remote strategies: e.g., remote triage via websites, call centers, etc;

		• LT_(long-term-solutions) Planning:<br>• LT1_Architecture, LT2_Engineering, LT3_Construction (builders), LTO_other hospital infrastructure planning
**RQ5**: What limitations were stated?	▪ Limitations stated	• Related to data<br>• Related to methods<br>• Not stated

Table S3. Search Results in PubMed

**PubMed Search strategy**		
Database: MEDLINE via PubMed		
Steps:<br>• Step1: #1<br>• Step2: #2<br>• Stpe3: #1 AND #2<br>• Step4: #1 AND #2 AND filters (Full text, English language, from 2019-2021)		
#1	Search: **"Hospital infrastructur*"[Title/Abstract] OR "hospital capacit*"[Title/Abstract] OR "hospital equipment*"[Title/Abstract] OR "hospital bed*"[Title/Abstract] OR "hospital plan*"[Title/Abstract]**	6,831 Results
#2	Search: **"COVID-19"[Title/Abstract] OR "Sars-Cov-2"[Title/Abstract] OR " Coronavirus"[Title/Abstract] OR "pandemic*"[Title/Abstract]**	243,785 Results
#1 AND #2	Search: **("Hospital infrastructur*"[Title/Abstract] OR "hospital capacit*"[Title/Abstract] OR "hospital equipment*"[Title/Abstract] OR "hospital bed*"[Title/Abstract] OR "hospital plan*"[Title/Abstract]) AND (COVID* OR Sars-Cov-2 OR Coronavirus OR pandemic*)**	562 Results
#1 AND #2 AND filters	Search: **("Hospital infrastructur*"[Title/Abstract] OR "hospital capacit*"[Title/Abstract] OR "hospital equipment*"[Title/Abstract] OR "hospital bed*"[Title/Abstract] OR "hospital plan*"[Title/Abstract]) AND (COVID* OR Sars-Cov-2 OR Coronavirus OR pandemic*)** Filters: **Full text, English, from 2019 - 2021**	489 Results

Table S4. Search Results in Scopus

**Scopus Search strategy**
Database: Scopus
Steps:<br>• Step1: #1<br>• Step2: #2

• Stpe3: #1 AND #2<br>• Step4: #1 AND #2 AND filters (Full text, English language, from 2019-2021)		
#1	TITLE-ABS-KEY ("Hospital infrastructur*" OR "hospital capacit*" OR "hospital equipment*" OR "hospital bed*" OR "hospital plan*")	53,063 document results
#2	TITLE-ABS-KEY (COVID-19 OR sars-cov-2 OR coronavirus OR pandemic*)	330,602 document results
#1 AND #2	(TITLE-ABS-KEY ("Hospital infrastructur*" OR "hospital capacit*" OR "hospital equipment*" OR "hospital bed*" OR "hospital plan*")) AND (TITLE-ABS-KEY (covid* OR sars-cov-2 OR coronavirus OR pandemic*))	1,504 document results
#1 AND #2 AND filters	(TITLE-ABS-KEY ("Hospital infrastructur*" OR "hospital capacit*" OR "hospital equipment*" OR "hospital bed*" OR "hospital plan*")) AND (TITLE-ABS-KEY (covid* OR sars-cov-2 OR coronavirus OR pandemic*)) AND (LIMIT-TO (PUBYEAR , 2021) OR LIMIT-TO (PUBYEAR , 2020) OR LIMIT-TO (PUBYEAR , 2019)) AND (LIMIT-TO (DOCTYPE , "ar")) AND (LIMIT-TO (LANGUAGE , "English"))	787 document results

Table S5. Search Results in Web of Science

**Web of Science Search strategy**		
Database: Web of Science		
Steps:<br>• Step1: #1<br>• Step2: #2<br>• Stpe3: #1 AND #2<br>• Step4: #1 AND #2 AND filters (Full text, English language, from 2019-2021)		
1	**(TI=("Hospital infrastructur*" OR "hospital capacit*" OR "hospital equipment*" OR "hospital bed*" OR "hospital plan*")) OR AB=("Hospital infrastructur*" OR "hospital capacit*" OR "hospital equipment*" OR "hospital bed*" OR "hospital plan*")**	4,816 results
2	**(TI=(COVID* OR Sars-Cov-2 OR Coronavirus OR pandemic*)) OR AB=(COVID* OR Sars-Cov-2 OR Coronavirus OR pandemic*)**	274,073 results
3	**#1 AND #2**	517 results
4	**#1 AND #2 and 2019 or 2020 or 2021 (Publication Years) and Articles (Document Types) and English (Languages)**	391 results

Table S6: All 106 studies included

**Study No**	**Accronym**	**Full reference**	**Type of publication**

1	Capolongo, S. et al. (2020)	Capolongo, S. et al. (2020) 'COVID-19 and Healthcare Facilities: a Decalogue of Design Strategies for Resilient Hospitals.', Acta bio-medica: Atenei Parmensis, 91(9-S), pp. 50–60. doi: 10.23750/abm.v91i9-S.10117.	Multicountry non-modelling
2	Louri, N. A. et al. (2021)	Louri, N. A. et al. (2021) 'Establishing a 130-Bed Field Intensive Care Unit to Prepare for COVID-19 in 7 Days in Bahrain Military Hospital', Disaster Medicine and Public Health Preparedness, 15(1), pp. e34–e43. doi: 10.1017/dmp.2020.297.	Singlecountry non-modelling
3	Lefrant, J.-Y. *et al.* (2020)	Lefrant, J.-Y. *et al.* (2020) 'A national healthcare response to intensive care bed requirements during the COVID-19 outbreak in France', *Anaesthesia Critical Care and Pain Medicine*, 39(6), pp. 709–715. doi: 10.1016/j.accpm.2020.09.007.	Singlecountry non-modelling
4	Fang, D. *et al.* (2020)	Fang, D. *et al.* (2020) 'Large-scale public venues as medical emergency sites in disasters: Lessons from COVID-19 and the use of Fangcang shelter hospitals in Wuhan, China', *BMJ Global Health*, 5(6), pp. 1–7. doi: 10.1136/bmjgh-2020-002815.	Singlecountry non-modelling
5	Arabi, Y. M. et al. (2021)	Arabi, Y. M. et al. (2021) 'How the COVID-19 pandemic will change the future of critical care', Intensive Care Medicine, 47(3), pp. 282–291. doi: 10.1007/s00134-021-06352-y.	Singlecountry non-modelling
6	Tadavarthy, S. N. et al. (2021)	Tadavarthy, S. N. et al. (2021) 'Developing and implementing an infection prevention and control program for a COVID-19 alternative care site in Philadelphia, PA', American Journal of Infection Control, 49(1), pp. 77–81. doi: 10.1016/j.ajic.2020.07.006	Singlecountry non-modelling
7	Hickey, S. et al. (2020)	Hickey, S. et al. (2020) 'Rapid deployment of an emergency department-intensive care unit for the COVID-19 pandemic', Clinical and Experimental Emergency Medicine, 7(4), pp. 319–325.	Singlecountry non-modelling
8	Emmanuel, U.et al. (2020)	Emmanuel, U., Osondu, E. D. and Kalu, K. C. (2020) 'Architectural design strategies for infection prevention and control (IPC) in health-care facilities: towards curbing the spread of Covid-19', *Journal of Environmental Health Science and Engineering*, 18(2), pp. 1699–1707.	Country-not-specified non-modelling
9	Akuamoa-Boateng, D. *et al.* (2020)	Akuamoa-Boateng, D. *et al.* (2020) 'Managing patient flows in radiation oncology during the COVID-19 pandemic: Reworking existing treatment designs to prevent infections at a German hot spot area University Hospital', *Strahlentherapie und Onkologie*, 196(12), pp. 1080–1085. doi: 10.1007/s00066-020-01698-6.	Singlecountry non-modelling

10	Whiteside, T. et al. (2020)	Whiteside, T. et al. (2020) 'Redesigning emergency department operations amidst a viral pandemic', American Journal of Emergency Medicine, 38(7), pp. 1448–1453. doi: 10.1016/j.ajem.2020.04.032.	Singlecountry non-modelling
11	Lacasa, L. et al. (2020)	Lacasa, L. et al. (2020) 'A flexible method for optimising sharing of healthcare resources and demand in the context of the COVID-19 pandemic', PLoS ONE, 15(10 October). doi: 10.1371/journal.pone.0241027.	Singlecountry non-modelling
12	Al-Dorzi, H. M. et al. (2021)	Al-Dorzi, H. M. et al. (2021) 'Managing critical care during COVID-19 pandemic: The experience of an ICU of a tertiary care hospital.', Journal of infection and public health, 14(11), pp. 1635–1641. doi: 10.1016/j.jiph.2021.09.018.	Singlecountry non-modelling
13	Nogués, X. et al. (2021)	Nogués, X. et al. (2021) 'Hospital-at-Home Expands Hospital Capacity During COVID-19 Pandemic.', Journal of the American Medical Directors Association, 22(5), pp. 939–942. doi: 10.1016/j.jamda.2021.01.077.	Singlecountry non-modelling
14	Kim, S. W. et al. (2020)	Kim, S. W. et al. (2020) 'A Brief Telephone Severity Scoring System and Therapeutic Living Centers Solved Acute Hospital-Bed Shortage during the COVID-19 Outbreak in Daegu, Korea.', Journal of Korean medical science, 35(15), pp. e152–e152.	Singlecountry non-modelling
15	Chen, Y. et al. (2020)	Chen, Y. et al. (2020) 'Emergency reconstruction of large general hospital under the perspective of new COVID-19 prevention and control', Wiener Klinische Wochenschrift, 132(21), pp. 677–684.	Singlecountry non-modelling
16	Witcher, T. R. (2020)	Witcher, T. R. (2020) 'Swift Support', Civil Engineering Magazine Archive, pp. 76–79. doi: 10.1061/ciegag.0001494.	Singlecountry non-modelling
17	Van Goethem, N. et al. (2020)	Van Goethem, N. et al. (2020) 'Rapid establishment of a national surveillance of COVID-19 hospitalizations in Belgium.', Archives of public health = Archives belges de sante publique, 78(1), p. 121. doi: 10.1186/s13690-020-00505-z.	Singlecountry non-modelling
18	Aziz, S. et al. (2020)	Aziz, S. et al. (2020) 'Managing ICU surge during the COVID-19 crisis: rapid guidelines', Intensive Care Medicine, 46(7), pp. 1303–1325. doi: 10.1007/s00134-020-06092-5.	Country-not-specified non-modelling
19	Bamias, G. et al. (2020)	Bamias, G. et al. (2020) 'The Greek Response to COVID-19: A True Success Story from an IBD Perspective', INFLAMMATORY BOWEL DISEASES, 26(8), pp. 1144–1148. doi: 10.1093/ibd/izaa143.	Singlecountry non-modelling
20	Borgen, I. et al. (2021)	Borgen, I. et al. (2021) 'From Hospital to Home: An Intensive Transitional Care Management Intervention for	Singlecountry non-modelling

		Patients with COVID-19.', Population health management, 24(1), pp. 27–34. doi: 10.1089/pop.2020.0178.	
21	Tan, Y. H. et al. (2021)	Tan, Y. H. et al. (2021) 'Application of a Machine Learning Algorithms in a Wrist-Wearable Sensor for Patient Health Monitoring during Autonomous Hospital Bed Transport.', Sensors (Basel, Switzerland), 21(17). doi: 10.3390/s21175711.	Singlecountry non-modelling
22	Hron, J. D. et al. (2020)	Hron, J. D. et al. (2020) 'Rapid Implementation of an Inpatient Telehealth Program during the COVID-19 Pandemic.', Applied clinical informatics, 11(3), pp. 452–459. doi: 10.1055/s-0040-1713635.	Singlecountry non-modelling
23	Luo, H. et al. (2020)	Luo, H. et al. (2020) 'Ultra-rapid delivery of specialty field hospitals to combat COVID-19: Lessons learned from the Leishenshan Hospital project in Wuhan', Automation in Construction, 119. doi: 10.1016/j.autcon.2020.103345.	Singlecountry non-modelling
24	Perondi, B. et al. (2020)	Perondi, B. et al. (2020) 'Setting up hospital care provision to patients with COVID-19: lessons learnt at a 2400-bed academic tertiary center in São Paulo, Brazil', Brazilian Journal of Infectious Diseases, 24(6), pp. 570–574. doi: 10.1016/j.bjid.2020.09.005.	Singlecountry non-modelling
25	Af Ugglas, B. et al. (2020)	Af Ugglas, B. et al. (2020) 'Emergency department crowding and hospital transformation during COVID-19, a retrospective, descriptive study of a university hospital in Stockholm, Sweden.', Scandinavian journal of trauma, resuscitation and emergency medicine, 28(1), p. 107. doi: 10.1186/s13049-020-00799-6.	Singlecountry non-modelling
26	Raith, E. P. et al. (2021)	Raith, E. P. et al. (2021) 'Repurposing a neurocritical care unit for the management of severely ill patients with COVID-19: A retrospective evaluation', Journal of Neurosurgical Anesthesiology, 33(1), pp. 77–81. doi: 10.1097/ANA.0000000000000727.	Singlecountry non-modelling
27	Brown, D. R. et al. (2020)	Brown, D. R. et al. (2020) 'Vancouver Convention Health Centre (COVID-19 Response): Planning, implementation, and four lessons learned', American journal of disaster medicine, 15(2), pp. 143–148. doi: 10.5055/ajdm.2020.0365.	Singlecountry non-modelling
28	Kim, M. *et al.* (2020)	Kim, M. *et al.* (2020) 'Lessons from a covid-19 hospital, republic of korea', *Bulletin of the World Health Organization*, 98(12), pp. 842–848. doi: 10.2471/BLT.20.261016.	Singlecountry non-modelling
29	Marcon, E. et al. (2020)	Marcon, E. et al. (2020) 'Schiavonia Hospital response to COVID-19 outbreak: a first single-center experience', Annali dell'Istituto Superiore di Sanita, 56(3), pp. 365–372. doi: 10.4415/ANN_20_03_15.	Singlecountry non-modelling

30	Joshi, M. and Kulkarni, M. (2021)	Joshi, M. and Kulkarni, M. (2021) 'Evaluation and Planning for a 250 Bedded COVID-19 Healthcare Infrastructure in City of Gurgaon, India', Hospital topics, 99(2), pp. 92–100. doi: 10.1080/00185868.2020.1859343.	Singlecountry non-modelling
31	Franke, G. et al. (2021)	Franke, G. et al. (2021) 'An automated room disinfection system using ozone is highly active against surrogates for SARS-CoV-2', Journal of Hospital Infection, 112, pp. 108–113. doi: 10.1016/j.jhin.2021.04.007.	Singlecountry non-modelling
32	Yang, Y., Kim, H. and Hwang, J. (2020)	Yang, Y., Kim, H. and Hwang, J. (2020) 'Quarantine Facility for Patients with COVID-19 with Mild Symptoms in Korea: Experience from Eighteen Residential Treatment Centers.', Journal of Korean medical science, 35(49), p. e429. doi: 10.3346/jkms.2020.35.e429.	Singlecountry non-modelling
33	Christen, P. *et al.* (2021)	Christen, P. *et al.* (2021) 'The J-IDEA Pandemic Planner: A Framework for Implementing Hospital Provision Interventions During the COVID-19 Pandemic.', *Medical care*, 59(5), pp. 371–378. doi: 10.1097/MLR.0000000000001502.	Multicountry non-modelling
34	Zhang, Y. et al. (2021)	Zhang, Y. et al. (2021) 'Wuhan mobile cabin hospital: A critical health policy at a critical time in China', Medicine, 100(3), p. e24077. doi: 10.1097/MD.0000000000024077.	Singlecountry non-modelling
35	Chen, S. et al. (2020)	Chen, S. et al. (2020) 'Fangcang shelter hospitals: a novel concept for responding to public health emergencies', The Lancet, 395(10232), pp. 1305–1314. doi: 10.1016/S0140-6736(20)30744-3.	Singlecountry non-modelling
36	Zhu, W. et al. (2020)	Zhu, W. et al. (2020) 'Establishing and Managing a Temporary Coronavirus Disease 2019 Specialty Hospital in Wuhan, China', Anesthesiology, 132(6), pp. 1339–1345. doi: 10.1097/ALN.0000000000003299.	Singlecountry non-modelling
37	Cai, Y. et al. (2020)	Cai, Y. et al. (2020) 'The effects of "Fangcang, Huoshenshan, and Leishenshan" hospitals and environmental factors on the mortality of COVID-19', PeerJ, 8, p. e9578.	Singlecountry non-modelling
38	Barasa, E. et al. (2020)	Barasa, E. W., Ouma, P. O. and Okiro, E. A. (2020) 'Assessing the hospital surge capacity of the Kenyan health system in the face of the COVID-19 pandemic.', PloS one, 15(7), p. e0236308. doi: 10.1371/journal.pone.0236308.	Singlecountry non-modelling
39	Poeran, J. et al. (2020)	Poeran, J. et al. (2020) 'Cancellation of Elective Surgery and Intensive Care Unit Capacity in New York State: A Retrospective Cohort Analysis.', Anesthesia and analgesia, 131(5), pp. 1337–1341. doi: 10.1213/ANE.0000000000005083.	Singlecountry non-modelling

40	Winkelmann, J. et al.(2022)	Winkelmann, J., Webb, E., Williams, G. A., Hernández-Quevedo, C., Maier, C. B., & Panteli, D. (2022). European countries' responses in ensuring sufficient physical infrastructure and workforce capacity during the first COVID-19 wave. Health Policy, 126(5), 362-372.	Multicountry non-modelling
41	López-Cheda, A. et al. (2021)	López-Cheda, A. et al. (2021) 'Estimating lengths-of-stay of hospitalized COVID-19 patients using a non-parametric model: A case study in Galicia (Spain)', Epidemiology and Infection. doi: 10.1017/S0950268821000959.	Singlecountry modelling
42	Bentout, S. et al. (2021)	Bentout, S. et al. (2021) 'Age-Structured Modeling of COVID-19 Epidemic in the USA, UAE and Algeria', Alexandria Engineering Journal, 60(1), pp. 401–411. doi: 10.1016/j.aej.2020.08.053.	Multicountry modelling
43	Rivera-Rodriguez, C. and Urdinola, B. P. (2020°	Rivera-Rodriguez, C. and Urdinola, B. P. (2020) 'Predicting Hospital Demand During the COVID-19 Outbreak in Bogotá, Colombia', Frontiers in Public Health, 8. doi: 10.3389/fpubh.2020.582706.	Singlecountry modelling
44	Gitto, S. et al. (2021)	Gitto, S. et al. (2021) 'Forecasting national and regional level intensive care unit bed demand during COVID-19: The case of Italy.', PloS one, 16(2), p. e0247726. doi: 10.1371/journal.pone.0247726.	Singlecountry modelling
45	Zhao, Ch. et al. (2020)	Zhao, Ch. et al. (2020) 'icumonitoring.ch: a platform for short-term forecasting of intensive care unit occupancy during the COVID-19 epidemic in Switzerland' Swiss Medical Weekly 150, doi: 10.4414/smw.2020.20277	Singlecountry modelling
46	Capistran, M. et al. (2021)	Capistran, M. et al. (2021) 'Forecasting hospital demand in metropolitan areas during the current COVID-19 pandemic and estimates of lockdown-induced 2nd waves ' PLoS ONE 16(1): e0245669. https://doi. org/10.1371/journal.pone.0245669	Singlecountry modelling
47	T. Mokhele et al. (2021)	T. Mokhele et al. (2021) 'Spatial analysis of perceived health system capability and actual health system capacity for covid-19 in south africa' Open Public Health J., vol. 14, no. 1, pp. 388–398, 2021, doi: 10.2174/1874944502114010388.	Singlecountry modelling
48	Moss, R. et al. (2020)	Moss, R. et al. (2020) 'Coronavirus Disease Model to Inform Transmission-Reducing Measures and Health System Preparedness, Australia' doi.org/10.3201/eid2612.202530	Singlecountry modelling
49	Tembine H. (2020)	Tembine H. (2020) 'COVID-19: Data-Driven Mean-Field-Type Game Perspective' Games 2020, 11, 51; doi:10.3390/g11040051	Multicountry modelling

50	Gel, ES., et al. (2020)	Gel, ES., et al. (2020) 'COVID-19 healthcare demand projections: Arizona' PLoS ONE 15(12): e0242588. https://doi.org/10.1371/journal. pone.0242588	Singlecountry modelling
51	Bayraktar Y., et al. (2020)	Bayraktar Y., et al. (2020) 'Role of the Health System in Combating Covid-19: Cross-Section Analysis and Artificial Neural Network Simulation for 124 Country Cases' doi.org/10.1080/19371918.2020.1856750	Multicountry modelling
52	Wells, C. M. et al (2021)	Wells, C. M. et al (2021) 'Tiered model of nurse staffing for critical care and emergency departments in the wake of a pandemic," J. Nurs. Adm., vol. 51, no. 2, pp. E1–E5, 2021, doi: 10.1097/NNA.0000000000000979.	Singlecountry modelling
53	Gambos, K., et al (2021).	Gambos, K., et al (2021). 'Translating Scientific Knowledge to Government Decision Makers Has Crucial Importance in the Management of the COVID-19 Pandemic' DOI: 10.1089/pop.2020.0159	Singlecountry modelling
54	Bhowmik T., et al. (2021)	Bhowmik T., et al. (2021) 'A comprehensive county level model to identify factors affecting hospital capacity and predict future hospital demand' doi.org/10.1038/s41598-021-02376-y	Singlecountry modelling
55	Stern, R. H. (2020)	Stern, R. H. (2020) 'Locally Informed Simulation to Predict Hospital Capacity Needs During the COVID-19 Pandemic.', Annals of internal medicine. United States, pp. 679–680. doi: 10.7326/L20-1061.	Singlecountry modelling
56	Smith, D. R. M. et al. (2020)	Smith, D. R. M. et al. (2020) 'Optimizing COVID-19 surveillance in long-term care facilities: a modelling study', BMC Medicine, 18(1). doi: 10.1186/s12916-020-01866-6.	Singlecountry modelling
57	Qian, Z.et al. (2021)	Qian, Z., Alaa, A. M. and van der Schaar, M. (2021) 'CPAS: the UK's national machine learning-based hospital capacity planning system for COVID-19', Machine Learning, 110(1), pp. 15–35. doi: 10.1007/s10994-020-05921-4.	Singlecountry modelling
58	de Barros Braga, M. et al. (2021)	de Barros Braga, M. et al. (2021) 'Artificial neural networks for short-term forecasting of cases, deaths, and hospital beds occupancy in the COVID-19 pandemic at the Brazilian Amazon', PLoS ONE, 16(3 March), p. e0248161. doi: 10.1371/journal.pone.0248161.	Singlecountry modelling
59	Guzzi, P. H., Tradigo, G. and Veltri, P. (2020)	Guzzi, P. H., Tradigo, G. and Veltri, P. (2020) 'Spatio-temporal resource mapping for intensive care units at regional level for COVID-19 emergency in Italy', International Journal of Environmental Research and Public Health, 17(10). doi: 10.3390/ijerph17103344.	Singlecountry modelling

60	Salles Neto, L. L. et al. (2020)	Salles Neto, L. L. et al. (2020) 'Forecast UTI: application for predicting intensive care unit beds in the context of the COVID-19 pandemic | Forecast UTI: aplicativo para previsão de leitos de unidades de terapia intensiva no contexto da pandemia de COVID-19', Epidemiologia e servicos de saude: revista do Sistema Unico de Saude do Brasil, 29(4), p. e2020391. doi: 10.5123/S1679-49742020000400023.	Singlecountry modelling
61	Barrett, K. et al. (2020)	Barrett, K. et al. (2020) 'Estimation of COVID-19-induced depletion of hospital resources in Ontario, Canada.', CMAJ: Canadian Medical Association journal = journal de l'Association medicale canadienne, 192(24), pp. E640–E646. doi: 10.1503/cmaj.200715	Singlecountry modelling
62	Iragorri, N. et al. (2020)	Iragorri, N. et al. (2020) 'Covid-19: Adaptation of a model to predict healthcare resource needs in valle del Cauca, Colombia [Covid-19: Adaptación de un modelo para predecir las necesidades de recursos de salud en el valle del cauca, Colombia]', Colombia Medica, 51(3), pp. 1–12. doi: 10.25100/cm.v51i3.e4534.	Singlecountry modelling
63	Akakba, A. and Lahmar, B. (2020)	Akakba, A. and Lahmar, B. (2020) 'The use of Geocoding for Home Healthcare Application and Management an Epidemic Situation. Case of COVID-19 Virus Outbreak', Geographica Pannonica, 24(4), pp. 285–293. doi: 10.5937/GP24-28062.	Singlecountry modelling
64	Presanis, A. M. et al. (2021)	Presanis, A. M. et al. (2021) 'Risk factors associated with severe hospital burden of COVID-19 disease in Regione Lombardia: a cohort study', BMC Infectious Diseases, 21(1). doi: 10.1186/s12879-021-06750-z.	Singlecountry modelling
63	Calabuig, J. M. et al. (2021)	Calabuig, J. M. et al. (2021) 'Modeling hospital resource management during the covid-19 pandemic: An experimental validation', Econometrics, 9(4). doi: 10.3390/econometrics9040038.	Singlecountry modelling
66	Faes, C., Hens, N. and Gilbert, M. (2021)	Faes, C., Hens, N. and Gilbert, M. (2021) 'On the timing of interventions to preserve hospital capacity: lessons to be learned from the Belgian SARS-CoV-2 pandemic in 2020.', Archives of public health = Archives belges de sante publique, p. 164. doi: 10.1186/s13690-021-00685-2.	Singlecountry modelling
67	Weissman, G. et al. (2020)	Weissman, G. et al. (2020) 'Locally Informed Simulation to Predict Hospital Capacity Needs During the COVID-19 Pandemic.',Ann Intern Med. 2020;173:21-28. doi:10.7326/M20-1260	Singlecountry modelling
68	Martin, C. et al. (2021)	Martin, C. et al. (2021) 'Construction of a demand and capacity model for intensive care and hospital ward	Singlecountry modelling

		beds, and mortality from COVID-19.' BMC Medical Informatics and Decision Making	
69	Berta, P. et al. (2020)	Berta, P. et al. (2020) 'A bivariate prediction approach for adapting the health care system response to the spread of COVID-19' PLoS ONE 15(10): e024015	Multicountry modelling
70	Lorenzen, S. et al. (2021)	Lorenzen, S. et al. (2021) 'Using machine learning for predicting intensive care unit resource use during the COVID-19 pandemic in Denmark' Nature - Scientific Reports doi./10.1038/s41598-021-98617-1	Singlecountry modelling
71	Shoukat, A. et al. (2020)	Shoukat, A. et al. (2020) 'Projecting demand for critical care beds during COVID-19 outbreaks in Canada' Research Health Services, CMAJ 2020 May 11;192:E489-96. doi: 10.1503/cmaj.200457; early-released April 8, 2020	Singlecountry modelling
72	Locey, K. et al. (2020)	Locey, K. et al. (2020) 'An interactive tool to forecast US hospital needs in the coronavirus 2019 pandemic' JAMIA Open, 3(4), 2020, 506–512 doi: 10.1093/jamiaopen/ooaa045	Singlecountry modelling
73	Bhandari, S. et al. (2020)	Bhandari, S. et al. (2020) 'Patient Flow Dynamics in Hospital Systems During Times of COVID-19: Cox Proportional Hazard Regression Analysis.' Front. Public Health 8:585850.doi: 10.3389/fpubh.2020.585850	Singlecountry modelling
74	McCabe, R. et al.(2021)	McCabe, R. et al. (2020/2021) 'Modelling intensive care unit capacity under different epidemiological scenarios of the COVID-19 pandemic in three Western European countries' International Journal of Epidemiology, 2021, 753–767 doi: 10.1093/ije/dyab034	Multicountry modelling
75	W. Knafo (2020)	W. Knafo, “COVID-19: Monitoring the propagation of the first waves of the pandemic,” 4Open, vol. 3, p. 5, 2020, doi: 10.1051/fopen/202000	Singlecountry modelling
76	Endres-Dighe S et al. (2021)	Endres-Dighe S et al. (2021) 'Lessons learned from the rapid development of a statewide simulation model for predicting COVID-19's impact on healthcare resources and capacity ' PLoS One. 2021 Nov 18;16(11):e0260310.doi: 10.1371/journal.pone.0260310. eCollection 2021.	Singlecountry modelling
77	Aborujilah, A., et al. (2021)	Aborujilah, A., Elsebaie, A.-E. F. M. and Mokhtar, S. A. (2021) ‘IoT MEMS: IoT-Based Paradigm for Medical Equipment Management Systems of ICUs in Light of COVID-19 Outbreak.’, IEEE access: practical innovations, open solutions, 9, pp. 131120–131133. doi: 10.1109/ACCESS.2021.3069255	Singlecountry modelling
78	Fort D et al. (2020)	Fort D et al. (2020) 'Locally Informed Modeling to Predict Hospital and Intensive Care Unit Capacity During the	Singlecountry modelling

		COVID-19 Epidemic 'Ochsner J. 2020 Fall; 20(3): 285–292.Published online Fall 2020. doi: 10.31486/toj.20.0073	
79	Ebinger J et al. (2021)	Ebinger J et al. (2021) 'A Machine Learning Algorithm Predicts Duration of hospitalization in COVID-19 patients' Intell Based Med. 2021; 5: 100035. Published online 2021 May 27. doi: 10.1016/j.ibmed.2021.100035	Singlecountry modelling
80	Verelst F. et al. (2020)	Verelst F. et al. (2020) 'Indications for healthcare surge capacity in European countries facing an exponential increase in coronavirus disease (COVID-19) cases, March 2020' Euro Surveill. 2020 Apr;25(13):2000323.doi: 10.2807/1560-7917.ES.2020.25.13.2000323.	Multicountry modelling
81	Li S. et al. (2020)	Li S. et al. (2020) 'Preliminary Assessment of the COVID-19 Outbreak Using 3-Staged Model e-ISHR'J. Shanghai Jiao Tong Univ. (Sci.), 2020, 25(2): 157-164	Singlecountry modelling
82	Beker R. et al. (2022)	Beker R. et al. (2022) 'Modeling COVID-19 hospital admissions and occupancy in the Netherlands 'Eur J Oper Res. 2022 Jan 5.doi: 10.1016/j.ejor.2021.12.044.	Singlecountry modelling
83	Wood R. et al. (2020)	Wood R. et al. (2020) 'COVID-19 scenario modelling for the mitigation of capacity-dependent deaths in intensive care ' Health Care Manag Sci. 2020 Sep;23(3):315-324. doi: 10.1007/s10729-020-09511-7.	Singlecountry modelling
84	Zhou W. et. al. (2020)	Zhou W. et. al. (2020) 'Impact of Hospital Bed Shortages on the Containment of COVID-19 in Wuhan' Int. J. Environ. Res. Public Health 2020, 17, 8560; doi:10.3390/ijerph17228560	Singlecountry modelling
85	Alqahtani F. et al. (2021)	Alqahtani F. et al. (2021) 'Bed Surge Capacity in Saudi Hospitals During the COVID-19 Pandemic' Disaster Med Public Health Prep . 2021 Apr 19;1-7.doi: 10.1017/dmp.2021.117.	Singlecountry modelling
86	Mishra S. et al. (2020)	Mishra S. et al. (2020) 'Estimated surge in hospital and intensive care admission because of the coronavirus disease 2019 pandemic in the Greater Toronto Area, Canada: a mathematical modelling study' CMAJ Open. 2020 Sep 22;8(3):E593-E604.doi: 10.9778/cmajo.20200093. Print Jul-Sep 2020.	Singlecountry modelling
87	McCabe R et al. (2021)	McCabe R et al. (2021) 'Modelling intensive care unit capacity under different epidemiological scenarios of the COVID-19 pandemic in three Western European countries 'International Journal of Epidemiology, 2021, 753–767 doi: 10.1093/ije/dyab034	Multicountry modelling
88	Chowdhury R et al. (2020)	Chowdhury R et al. (2020)' Dynamic interventions to control COVID-19 pandemic: a multivariate prediction	Multicountry modelling

		modelling study comparing 16 worldwide countries' European Journal of Epidemiology (2020) 35:389–399 https://doi.org/10.1007/s10654-020-00649-w	
89	Deschepper M et al. (2021)	Deschepper M et al. (2021) 'Prediction of hospital bed capacity during the COVID– 19 pandemic' BMC Health Serv Res. 2021 May 18;21(1):468.doi: 10.1186/s12913-021-06492-3	Singlecountry modelling
90	Chin V et al. (2020	Chin V et al. (2020) 'A case study in model failure? COVID-19 daily deaths and ICU bed utilisation predictions in New York state'European Journal of Epidemiology (2020) 35:733–742 https://doi.org/10.1007/s10654-020-00669-6	Singlecountry modelling
91	Goic M et al. (2021)	Goic M et al. (2021) COVID-19: Short-term forecast of ICU beds in times of crisis ' PLoS One. 2021 Jan 13;16(1):e0245272.doi: 10.1371/journal.pone.0245272. eCollection 2021.	Singlecountry modelling
92	Alqahtani R. T. (2021)	Alqahtani, R. T. (2021) 'Mathematical model of SIR epidemic system (COVID-19) with fractional derivative: stability and numerical analysis.' Adv. Differ. equations, vol. 2021, no. 1, p. 2, 2021, doi: 10.1186/s13662-020-03192-w.	Singlecountry modelling
93	Baas S. et al. (2021)	Baas S. et al. (2021) 'Real-time forecasting of COVID-19 bed occupancy in wards and Intensive Care Units'Health Care Manag Sci. 2021 Jun;24(2):402-419.doi: 10.1007/s10729-021-09553-5. Epub 2021 Mar 25.	Singlecountry modelling
94	Cheng FY et al. (2020)	Cheng FY et al. (2020)'Using Machine Learning to Predict ICU Transfer in Hospitalized COVID-19 Patients' J Clin Med. 2020 Jun 1;9(6):1668.doi: 10.3390/jcm9061668.	Singlecountry modelling
95	Manca D et al. (2020)	Manca D et al. (2020)'A simplified math approach to predict ICU beds and mortality rate for hospital emergency planning under Covid-19 pandemic' Comput Chem Eng. 2020 Sep 2;140:106945.doi: 10.1016/j.compchemeng.2020.106945. Epub 2020 Jun 4.	Singlecountry modelling
96	I. COVID and C. J. L. Murray (2020)	I. COVID and C. J. L. Murray, "Forecasting the impact of the first wave of the COVID-19 pandemic on hospital demand and deaths for the USA and European Economic Area countries," MedRxiv, vol. 1, pp. 1–26, 2020.	Multicountry modelling
97	Vekaria, B. et al. (2021)	Vekaria, B. et al. (2021) 'Hospital length of stay for COVID-19 patients: Data-driven methods for forward planning.', BMC infectious diseases, 21(1), p. 700. doi: 10.1186/s12879-021-06371-6.	Singlecountry modelling
98	Rees, E. M. et al. (2020)	Rees, E. M. et al. (2020) 'COVID-19 length of hospital stay: a systematic review and data synthesis.', BMC medicine, 18(1), p. 270. doi: 10.1186/s12916-020-01726-3.	Singlecountry Systematic Review

99	Ravaghi, H. et al. (2020)	Ravaghi, H. et al. (2020) 'Models and methods for determining the optimal number of beds in hospitals and regions: a systematic scoping review', BMC health services research, 20(1), pp. 1–13.	No-country-stated Scoping Review
100	Sim, S. S. et al. (2021)	Sim, S. S. et al. (2021) 'Digital technology for amd management in the post-covid-19 new normal', Asia-Pacific Journal of Ophthalmology, 10(1), pp. 39–48.	Singlecountry Literature Review
101	McCabe, R. et al. (2020)	McCabe, R. et al. (2020) 'Adapting hospital capacity to meet changing demands during the COVID-19 pandemic.', BMC medicine, 18(1), pp. 1–12	Singlecountry dataset Analysis and Literature Review
102	Klein, M. et al. (2020)	Klein, M. et al. (2020) 'COVID-19 Models for Hospital Surge Capacity Planning: A Systematic Review' Disaster Medicine and Public Health Preparednes, doi 10.1017/dmp.2020.332	Singlecountry Systematic Review
103	Cancino et al. (2020)	Cancino A, Castillo C, De Wolff T, Gajardo P, Lecaros R, Munoz C, et al. Report# 4: Estimation of maximal ICU beds demand for COVID-19 outbreak in some Chilean regions and the effects of different mitigation strategies. Technical report, CMM-AM2V-CEPS, 03 2020. URL: http://covid-19.cmm.uchile.cl;2020.	Singlecountry technical report
104	Van de Voorde et al. (2020)	Van de Voorde C, Lefèvre M, Mistiaen P, Detollenaere J, Kohn L, Van den<br>Heede K. Assessing the management of hospital surge capacity in the firstwave of the COVID-19 pandemic in Belgium. In: KCE reports. Brussels: Belgian Health Care Knowledge Centre (KCE); 2020.	Singlecountry technical report
105	Sagan et al. (2021)	Sagan A, Webb A, Azzopardi-Muscat N, de la Mata I, McKee M, Figueras J (ed). Health systems resilience during COVID-19: Lessons for building back better. European Observatory on Health System and Policies, WHO, 2021	Multicountry technical report
106	Rentschler et al. (2021)	Rentschler, Jun; Klaiber, Christoph; Tariverdi, Mersedeh; Desjonqueres, Chloe; Mercadante, Jared. 2021. Frontline: Preparing Healthcare Systems for Shocks from Disasters to Pandemics. World Bank, Washington, DC. © World Bank. https://openknowledge.worldbank.org/handle/10986/35429	Multicountry technical report

Table S7. Results of five included review studies.

Author(s)	Publication date	Type of review	Review objective	Number of sudies included	Adaptations proposed during COVID-19	Applicability to long-term/ planning

Rees, E. M. et al.[15]	2020/09	Systematic Review	Reviewing early evidence on the length of stay (LoS) of patients with COVID-19 in hospitals and in ICUs.	52	Monitoring the distributions of total hospital and ICU LoS.	Important to project future demand and make early estimates of how long patients need to stay in hospitals.
Ravaghi, H. et al.[17]	2020/03	Scoping Review	Identifying and characterizing existing models and methods for determining the required number of beds at the hospital and regional levels, as well as comparing their benefits and drawbacks.	23	Authors suggested considering alternative approaches to planning hospital capacity, like care pathways, to fix the limitations of "bed numbers".	Decision-making about the optimal number of hospital beds at the hospital or regional levels.
Sim, S. S. et al.[121]	2021/02	Literature Review	The review looked at how pressures created by COVID-19 in health systems and hospital infrastructures have encouraged the acceptance and speed of adoption of digitalization.	15	Implementation of artificial intelligence and “virtual clinics” in screening, diagnosis, monitoring, and treatment; home monitoring devices in self-monitoring approaches.	Digitalization in healthcare delivery (in the post-COVID-19 new normal where digital platforms may be routine, standard, and expected in healthcare delivery).
McCabe, R. et al.[14]	2020/10	Analysis of National Health Service (NHS) datasets and Literature Review	Estimating hospital care capacity before the pandemic and quantifying the impact of interventions (cancellation of elective surgery, field hospitals, use of private hospitals,	37	Cancellation of elective surgeries; set-up of field hospitals; use of private hospitals; redeployment of former healthcare staff and deployment of newly qualified and final year nursing and medical students.	Authors developed a model to quantify hospital capacity for general and acute and critical care considering three crucial resources: staff, beds, and ventilators.

			deployment of former medical staff and deployment of newly qualified medical staff) for treatment COVID-19 patients.			
Klein, M. et al.[16]	2020/10	Systematic Review	Highlighting the models that project both caseload and hospital capacity requirements.	6	Using projection models to manage hospital capacity: including length of stay (LOS), occupancy, mortality, and ventilator capacity.	Adoption of models that help with both caseload projection and hospital capacity management
